# Disparities in tissue-based biomarker testing among US Medicare beneficiaries with prostate cancer

**DOI:** 10.1093/jncics/pkaf051

**Published:** 2025-05-16

**Authors:** Stephan M Korn, Zhiyu Qian, Hanna Zurl, Andrea Piccolini, Klara K Pohl, Stuart Lipsitz, Jianyi Zhang, Adam S Kibel, Caroline M Moore, Huma Q Rana, Kerry L Kilbridge, Shahrokh F Shariat, Stacy Loeb, Quoc-Dien Trinh, Alexander P Cole

**Affiliations:** Department of Urology, Medical University of Vienna, Vienna, Austria; Center for Surgery and Public Health, Brigham and Women’s Hospital, Harvard Medical School, Boston, MA, United States; Center for Surgery and Public Health, Brigham and Women’s Hospital, Harvard Medical School, Boston, MA, United States; Department of Urology, Brigham and Women’s Hospital, Harvard Medical School, Boston, MA, United States; Center for Surgery and Public Health, Brigham and Women’s Hospital, Harvard Medical School, Boston, MA, United States; Department of Urology, Medical University of Graz, Graz, Austria; Center for Surgery and Public Health, Brigham and Women’s Hospital, Harvard Medical School, Boston, MA, United States; Department of Urology, Humanitas Clinical and Research Hospital, Milan, Italy; Center for Surgery and Public Health, Brigham and Women’s Hospital, Harvard Medical School, Boston, MA, United States; Department of Urology, Medical University of Graz, Graz, Austria; Center for Surgery and Public Health, Brigham and Women’s Hospital, Harvard Medical School, Boston, MA, United States; Center for Surgery and Public Health, Brigham and Women’s Hospital, Harvard Medical School, Boston, MA, United States; Department of Urology, Brigham and Women’s Hospital, Harvard Medical School, Boston, MA, United States; Division of Surgery and Interventional Science, University College London, London, United Kingdom; Division of Cancer Genetics and Prevention, Dana-Farber Cancer Institute, Harvard Medical School, Boston, MA, United States; Department of Medical Oncology, Dana-Farber Cancer Institute, Harvard Medical School, Boston, MA, United States; Lank Center for Genitourinary Oncology, Dana-Farber Cancer Institute, Boston, MA, United States; Department of Urology, Medical University of Vienna, Vienna, Austria; Karl Landsteiner Gesellschaft, Institute of Urology and Andrology, Vienna, Austria; Department of Urology, Second Faculty of Medicine, Charles University, Prague, Czech Republic; Division of Urology, Hourani Center for Applied Scientific Research, Al-Ahliyya Amman University, Amman, Jordan; Department of Urology, Weill Cornell Medical College, New York, NY, United States; Department of Urology, University of Texas Southwestern Medical Centre, Dallas, TX, United States; Department of Urology and Population Health, New York University Langone Health and Manhattan Veterans Affairs, New York, NY, United States; Department of Urology, University of Pittsburgh, Pittsburgh, PA, United States; Center for Surgery and Public Health, Brigham and Women’s Hospital, Harvard Medical School, Boston, MA, United States; Department of Urology, Brigham and Women’s Hospital, Harvard Medical School, Boston, MA, United States

## Abstract

**Background:**

Personalized therapeutic approaches for localized prostate cancer have evolved significantly, with tissue-based biomarker tests supplementing traditional risk stratification tools. However, national testing patterns and geographic variability remain limited a decade after coverage implementation. We aimed to assess current nationwide utilization and urban-rural differences in tissue-based biomarker testing.

**Methods:**

Using full Medicare claims data, we retrospectively identified patients with newly diagnosed prostate cancer and tissue-based biomarker testing claims from 2019 to 2023. Patients' county of residence was categorized as metro, urban, or rural. Regional testing rates were further assessed across hospital referral regions. A multivariable logistic regression model was performed to assess the effect of residence on test receipt.

**Results:**

Our final cohort included 749 202 patients, of whom 79.5% lived in metro, 11.4% in urban and 8.00% in rural counties. Overall, 86 908 (11.6%) patients underwent tissue-based biomarker tests. Hospital referral region-level testing rates ranged from 2.4% to 42.7%. Rural patients were 18% less likely to undergo testing compared to metro patients (odds ratio [OR] 0.82, 95% CI = 0.73 to 0.91). Independently, the odds of undergoing testing were lower among Black (OR 0.82, 95% CI = 0.77 to 0.88) and Hispanic patients (OR 0.80, 95% CI = 0.73 to 0.88) compared to White patients.

**Conclusion:**

This study reveals high geographic variability in tissue-based biomarker testing for prostate cancer. Further, Black and Hispanic patients were less likely to receive testing. Our findings highlight regional practice variation in the use of advanced, not routinely recommended tests and underscore the need to minimize disparities in diagnostic access.

## Introduction

While prostate cancer remains the most prevalent solid organ cancer in males,^1^ most will present with localized disease.^2^ Standard treatment options for localized prostate cancer include active surveillance, radical prostatectomy, or radiation therapy. A central challenge in the choice of treatment is to balance the overtreatment of indolent disease with the undertreatment of more aggressive cancers. Rural patients may be particularly affected by these challenges. For instance, appropriate candidates for active surveillance are less likely to receive this treatment option in rural areas.^3^ Addressing inequities is important, as eliminating disparities in access to care could lead to comparable longtime outcomes.^4^^,^^5^

Tissue-based biomarker tests—examples of which include Prolaris, Decipher, Oncotype DX, and Promark—have emerged as tools for risk assessment in prostate cancer management.^6^ Based on individual genetic expression and protein biomarker information, these tests were developed to calculate risk scores prognostic for adverse pathology and long-term clinical outcomes.^27^^,^^40^ While not routinely recommended due to limited evidence of clinical impact,^37^^,^^38^ tissue-based biomarker tests could provide information to support treatment decisions, such as active surveillance and multimodal therapy.^6^^,^^7^ Since 2015, Medicare coverage has begun for commercially available tests.^8^^,^^9^ However, healthcare innovations traditionally tend to be implemented first in urban centers before reaching rural regions.^10^^,^^11^ Further, regional practice-level, non-clinical factors may influence the use of emerging diagnostic technologies.^16^ Some diagnostic modalities, such as advanced imaging procedures, require expensive onsite investments and patient travel to facilities, and may therefore remain less available in rural and low-resource settings.^12^^,^^13^ Unlike these tests for risk stratification, tissue-based biomarker tests require fewer resources.^14^^,^^15^

However, real-world utilization as well as the impact of residence and other patient characteristics on the performance of these tests remain scarce. Given their implementation and coverage starting a decade ago, along with observed delays in rural adoption, geographic variations in utilization of tissue-based biomarker test modalities may exist. In this setting, we sought to assess national patterns of the use of tissue-based risk score generating tests based on patient’s geographic region and sociodemographic characteristics, and specifically, whether utilization of tissue-based biomarker tests was different in rural settings.

## Methods

### Data source

For this nationwide analysis, we utilized full Medicare claims data between January 2019 and December 2023. The federal health insurance program Medicare primarily covers individuals aged 65 years or older, while also extending benefits to certain younger adults with disabilities. Medicare data cover comprehensive information on diagnoses and the use of health care services by beneficiaries. It also includes demographic factors such as beneficiaries’ age, geographic location, race/ethnicity, and gender, but lacks clinicopathologic data. While SEER-Medicare linked data would provide clinicopathological information, they cover only a limited number of registries and exclude significant rural regions. Given these limitations for studying geographic disparities and rural-urban differences,^17^^,^^18^ we chose to analyze complete Medicare claims data for our analysis.

Patient data were linked to hospital referral regions from the Dartmouth Atlas using a previously described methodology.^19^ Independent from any rural/urban classification, the Dartmouth Atlas Hospital Referral Regions are geographic areas defined by regional hospital care patterns and are a commonly used tool to measure regional variability in care.^20^^,^^21^

### Study cohort

Patients newly diagnosed with prostate cancer from January 2019 to December 2023, identified by the International Classification of Diseases, 10th revision, Clinical Modification topography code C61, were included in the analysis. We first examined 2018 claims to identify and exclude patients with previously diagnosed prostate cancer. Additional exclusion criteria encompassed beneficiaries below 66 years of age at diagnosis, those with Medicare enrollment duration less than 12 months, and individuals with missing geographic identifiers (Figure S1).

### Dependent variable

The primary endpoint was defined as the receipt of tissue-based biomarker testing from 2019 through 2023 following prostate cancer diagnosis. We included the commercially available Prolaris, Decipher, Oncotype DX, and Promark tissue-based biomarker tests in our analysis. To ensure our analysis captured all commercially available tests, we incorporated both specific and general Healthcare Common Procedure Coding System codes for prostate cancer tissue-based biomarker tests, as certain commercially available assays, such as Promark, lack dedicated codes. This approach enabled us to identify all established commercial tests, while acknowledging that some miscoded tests or overlapping test types beyond our primary focus may also have been included. Table S1 lists the codes used to define our endpoint.

### Predictor variable

We linked patients’ designed Federal Information Processing System codes to the 2023 Rural-Urban Continuum Codes (RUCC) published by the US Department of Agriculture.^22^ Based on the 2023 boundaries established by the Office of Management and Budget, RUCC classifies counties into metro and non-metro areas. Following published literature, non-metro counties were subdivided into urban and rural counties (Table S2).^3^^,^^23^ The classification distinguishes metro counties with large cities, urban counties with mid-sized and suburban towns, and rural counties with dispersed populations.

### Covariates

We identified the following covariates from Medicare claims data: age at diagnosis, race (by Medicare race code classified as “Unknown,” “White,” “Black,” “Other,” “Asian,” “Hispanic,” and “North American Native”), year of prostate cancer diagnosis, dual Medicare/Medicaid eligibility, disability as the original reason for Medicare eligibility, number of chronic conditions excluding prostate cancer, and US Census regions. Variables are categorized as shown in Table 1.

**Table 1.** Baseline characteristics of patients in metro, rural, and urban counties of residence.

	Overall, *n* (%)	Metro, *n* (%)	Urban, *n* (%)	Rural, *n* (%)	Unknown, *n* (%)
	*n* = 749 202 (100%)	*n* = 595 843 (79.5%)^a^	*n* = 85 222 (11.4%)^a^	*n* = 59 823 (8.0%)^a^	*n* = 8 314 (1.1%)^a^
Demographics and health status					
**Age** **group**					
66-70	191 882 (25.6%)	151 638 (25.5%)	22 503 (26.4%)	15 848 (26.5%)	1 893 (22.8%)
71-75	213 689 (28.5%)	170 624 (28.6%)	24 306 (28.5%)	16 618 (27.8%)	2 141 (25.8%)
76-80	157 167 (21.0%)	125 209 (21.0%)	17 602 (20.7%)	12 588 (21.0%)	1 768 (21.3%)
≥81	186 464 (24.9%)	148 372 (24.9%)	20 811 (24.4%)	14 769 (24.7%)	2 512 (30.2%)
**Race/Ethnicity**					
White	629 360 (84.0%)	492 187 (82.6%)	76 027 (89.2%)	53 981 (90.2%)	7 165 (86.2%)
Black	64 800 (8.7%)	56 170 (9.4%)	5 039 (5.9%)	3 132 (5.2%)	459 (5.5%)
Hispanic	7 374 (1.0%)	6 679 (1.1%)	415 (0.5%)	213 (0.4%)	67 (0.8%)
Asian	8 737 (1.2%)	8 425 (1.4%)	185 (0.2%)	48 (0.1%)	79 (1.0%)
Other	14 872 (2.0%)	12 302 (2.1%)	1 370 (1.6%)	1 070 (1.8%)	130 (1.6%)
Unknown	24 059 (3.2%)	20 080 (3.4%)	2 186 (2.6%)	1 379 (2.3%)	414 (5.0%)
**US** **Region**					
South	271 397 (36.2%)	215 474 (36.2%)	32 189 (37.8%)	23 582 (39.4%)	152 (1.8%)
West	157 245 (21.0%)	131 093 (22.0%)	16 435 (19.3%)	9 583 (16.0%)	134 (1.6%)
Midwest	157 345 (21.0%)	110 709 (18.6%)	24 489 (28.7%)	22 064 (36.9%)	83 (1.0%)
Northeast	163 215 (21.8%)	138 567 (23.3%)	12 109 (14.2%)	4 594 (7.7%)	7 945 (95.6%)
**Dual eligibility** **for** **Medicare/Medicaid**					
Yes	50 101 (6.7%)	39 393 (6.6%)	5 259 (6.2%)	4 492 (7.5%)	957 (11.5%)
No	699 101 (93.31%)	556 450 (93.4%)	79 963 (93.8%)	55 331 (92.5%)	7 357 (88.5%)
**Disability as the original reason for Medicare** **entitlement**					
Yes	63 618 (8.5%)	47 138 (7.9%)	8 810 (10.3%)	7 049 (11.8%)	621 (7.5%)
No	685 584 (91.5%)	548 705 (92.1%)	76 412 (89.7%)	52 774 (88.2%)	7 693 (92.5%)
**Year of prostate cancer** **diagnosis**					
2019	212 466 (28.4%)	168 603 (28.3%)	24 431 (28.7%)	16 953 (28.3%)	2 479 (29.8%)
2020	144 511 (19.3%)	114 635 (19.2%)	16 548 (19.4%)	11 768 (19.7%)	1 560 (18.8%)
2021	138 908 (18.5%)	110 638 (18.6%)	15 722 (18.5%)	10 988 (18.4%)	1 560 (18.8%)
2022	130 269 (17.4%)	103 904 (17.4%)	14 622 (17.2%)	10 312 (17.2%)	1 431 (17.2%)
2023	123 048 (16.4%)	98 063 (16.5%)	13 899 (16.3%)	9 802 (16.4%)	1 284 (15.4%)
**Number of chronic** **conditions**					
0	48 639 (6.5%)	37 716 (6.3%)	5 869 (6.9%)	4 584 (7.7%)	470 (5.7%)
1	41 652 (5.6%)	31 764 (5.3%)	5 293 (6.2%)	4 173 (7.0%)	422 (5.1%)
2	66 908 (8.9%)	51 801 (8.7%)	8 263 (9.7%)	6 217 (10.4%)	627 (7.5%)
≥3	592 003 (79.0%)	474 562 (79.7%)	65 797 (77.21%)	44 849 (75.0%)	6 795 (81.7%)

a Percentages are given in column percentages unless indicated by “a” for row percentages.

### Statistical analysis

Proportions of the distribution in baseline categorical demographic variables were calculated in overall, metro, rural, and urban patients. The proportions of eligible beneficiaries who received tissue-based biomarker tests were compared in metro, rural and urban patients using the chi-square test.

To evaluate differences between patients with and without tissue-based biomarker tests, chi-square tests were performed for categorical covariates. A patient-level multivariable logistic regression model was conducted. The model included the main predictor variable (metro, urban and rural residence) and was adjusted for our predefined patient level covariates. The model was clustered by hospital referral regions. This approach ensured accurate adjustment of standard errors in the logistic regression model. A secondary analysis included an interaction term between residency (metro, urban, and rural) and years of diagnosis (2019-2023).

Analyses were conducted using SAS Enterprise Guide 7.1, Cary, North Carolina.

## Results

Our cohort consisted of 749 202 beneficiaries with newly diagnosed prostate cancer between 2019 and 2023. Of them, 595 843 (79.5%) lived in metro counties, whereas 85 222 (11.4%) beneficiaries lived in urban counties, and 59 823 (8.0%) beneficiaries lived in rural counties. Across metro, urban, and rural areas the majority of patients had at least three comorbidities and were predominantly white. Sociodemographic characteristics of beneficiaries in the total study population and in metro, urban, and rural counties are summarized in Table 1.

Overall, 86 908 (11.6%) patients received a tissue-based biomarker test within the study period. Based on county of residence 12.0% (95% CI = 12.0% to 12.1%) of metro patients, 9.8% (95% CI = 9.6% to 10.0%) of urban patients and 10.0% (95% CI = 9.8% to 10.3%) of rural patients received tissue-based biomarker tests, *P* < .0001. Median age was lower in patients who received biomarker tests compared to those who did not (73 years, IQR 69-77, and 75 years, IQR 71-81, respectively, *P* < .0001). The proportion of patients receiving a test increased in more recent years of diagnosis, from 6.9% in 2019 to 16.4% in 2023 (*P* < .0001). Test use was higher among White patients (11.7%) compared to all other racial groups (10.2% in Asian, 10.0% in Other, 9.6% in Black, and 8.3% in Hispanic patients), *P* < .0001. Testing rates were higher in non-dually Medicare/Medicaid eligible patients, and in Medicare non-disability enrollees. Detailed results are given in Table 2.

**Table 2.** Baseline characteristics of patients with and without a tissue-based biomarker test.^a^

		Tissue-based biomarker tests		
	Overall	*Tissue-based biomarker tests*	*No tissue-based biomarker tests*	*P^b^*
	*n* = 749 202 (100%)	*n* = 86 908 (11.60%)	*n* = 662 294 (88.40%)	
**Residency**				
Metro	595 843 (100%)	71 688 (12.0%)	524 155 (88.0%)	<.0001
Urban	85 222 (100%)	8 337 (9.8%)	76 885 (90.2%)	
Rural	59 823 (100%)	6 000 (10.0%)	53 823 (90.0%)	
Unknown	8 314 (100%)	883 (10.6%)	7431 (89.4%)	
**Age** **group**				
66-70 years	191 882 (100%)	28 845 (15.0%)	163 037 (85.0%)	<.0001
71-75 years	213 689 (100%)	30 019 (14.1%)	183 670 (86.0%)	
76-80 years	157 167 (100%)	17 790 (11.3%)	139 377 (88.7%)	
≥81 years	186 464 (100%)	10 254 (5.5%)	176 210 (94.5%)	
**Race/Ethnicity**				
White	629 360 (100%)	73 751 (11.7%)	555 609 (88.3%)	<.0001
Black	64 800 (100%)	6 215 (9.6%)	58 585 (90.4%)	
Hispanic	7 374 (100%)	609 (8.3%)	6 765 (91.7%)	
Asian	8 737 (100%)	888 (10.2%)	7 849 (89.8%)	
Other	14 872 (100%)	1 493 (10.0%)	13 379 (90.0%)	
Unknown	24 059 (100%)	3 952 (16.4%)	20 107 (83.6%)	
**US** **Region**				
South	271 397 (100%)	32 608 (12.0%)	238 789 (88.0%)	<.0001
West	157 245 (100%)	19 016 (12.1%)	138 229 (87.9%)	
Midwest	157 345 (100%)	17 652 (11.2%)	139 693 (88.8%)	
Northeast	163 215 (100%)	17 632 (10.8%)	145 583 (89.2%)	
**Dual eligibility for** **Medicaid**				
Yes	50 101 (100%)	3 657 (7.3%)	46 444 (92.7%)	<.0001
No	699 101 (100%)	83 251 (11.9%)	615 850 (88.1%)	
**Disability as the original reason for Medicare** **entitlement**				
Yes	63 618 (100%)	5 605 (8.8%)	58 013 (91.2%)	<.0001
No	685 584 (100%)	81 303 (11.9%)	604 281 (88.1%)	
**Year of prostate cancer** **diagnosis**				
2019	212 466 (100%)	14 721 (6.9%)	197 745 (93.1%)	<.0001
2020	144 511 (100%)	13 663 (9.5%)	130 848 (90.6%)	
2021	138 908 (100%)	17 507 (12.6%)	121 401 (87.4%)	
2022	130 269 (100%)	20 868 (16.0%)	109 401 (84.0%)	
2023	123 048 (100%)	20 149 (16.4%)	102 899 (83.6%)	
**Number of chronic** **conditions**				
0	48 639 (100%)	6 696 (13.8%)	41 943 (86.2%)	<.0001
1	41 652 (100%)	5 280 (12.7%)	36 372 (87.3%)	
2	66 908 (100%)	8 610 (12.9%)	58 298 (87.1%)	
≥3	592 003 (100%)	66 322 (11.2%)	525 681 (88.8%)	

a Percentages are given in row percentages.

b Chi-Square test was used to test significance.

**Figure 1.**
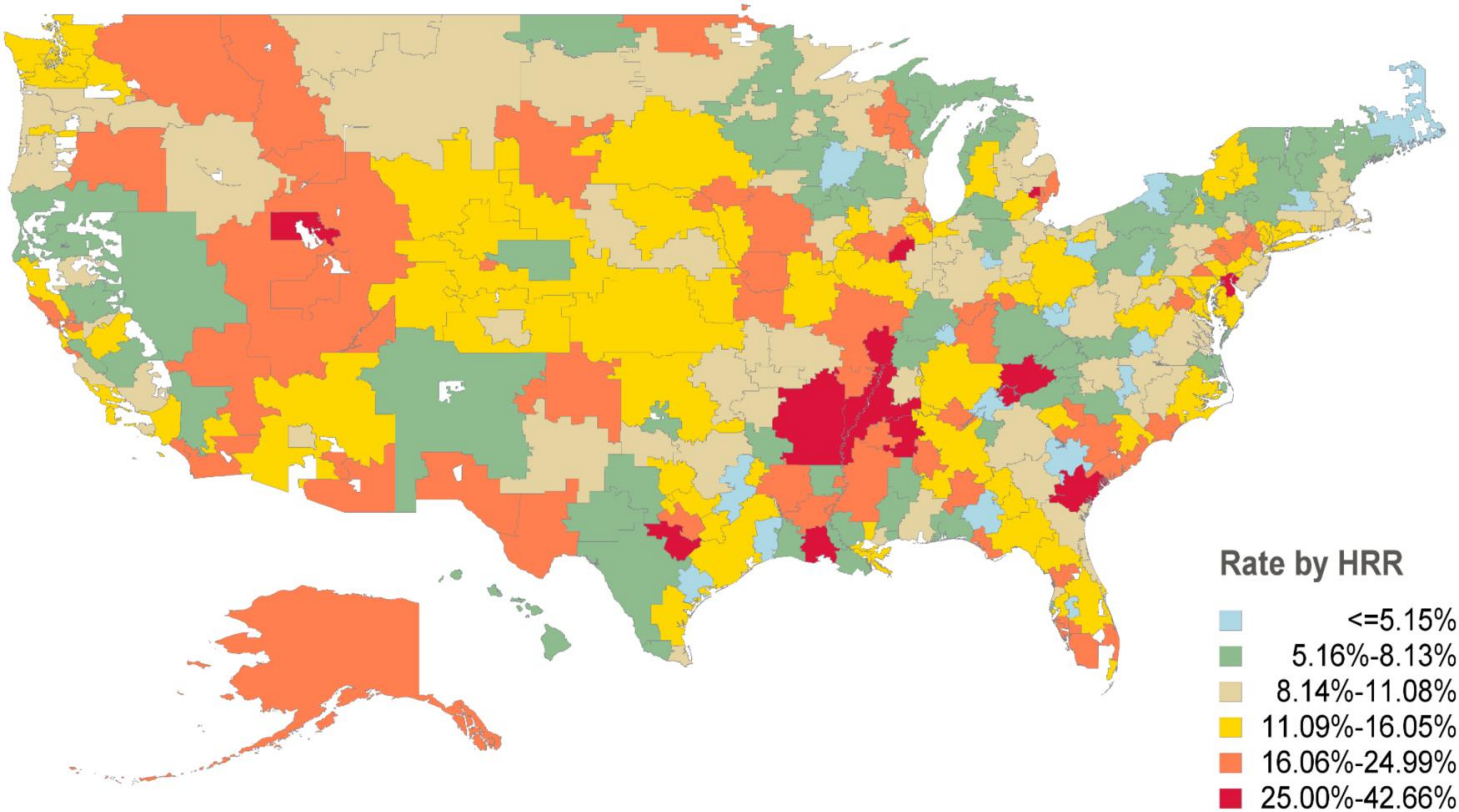
Heatmap of testing rates in individual hospital referral regions.

Testing utilization ranged from a minimum of 2.4% to a maximum of 42.7% (median testing rate: 11.0%, IQR 8.0% to 16.0%) across 306 hospital referral regions in patients with newly diagnosed prostate cancer. A heatmap of tissue-based biomarker tests, showing the frequency of their utilization relative to newly diagnosed prostate cancer cases in individual HRRs, is provided in Figure 1.

The patient-level multivariable regression model, clustered by hospital referral regions, demonstrated significantly lower odds of tissue-based biomarker tests in both rural and urban areas compared to metro areas. Using metro counties (largest population centers) as the reference, rural patients were 18% less likely to receive tissue-based biomarker tests (OR 0.82, 95% CI = 0.73 to 0.91) and urban patients (mid-sized population centers) were 22% less likely to undergo testing (OR 0.78, 95% CI = 0.72 to 0.85).

Independently, Black (OR 0.82, 95% CI = 0.77 to 0.88) and Hispanic (OR 0.80, 95% CI = 0.73 to 0.88) patients had lower odds of tissue-based biomarker tests compared to white beneficiaries. Increasing age was also associated with lower testing odds. Testing odds increased with later diagnosis years (2019-2023), more chronic conditions, non-dual Medicare/Medicaid status, and non-disability Medicare enrollment. Table 3 summarizes patient level predictors of receiving tissue-based biomarker tests. In a secondary analysis, we found no significant interaction (*P* = .82) between residence (metro, urban, rural) and year of diagnosis (2019-2023). In this model with interaction, rural and urban county patients were consistently less likely to undergo testing compared to metro counties across the years (Figure S2). This result agrees with the original multivariate logistic regression model without the interaction term (Table 3), which also found that patients from rural and urban counties were less likely to undergo testing compared to those from metro counties.

**Table 3.** Hospital referral region-clustered multivariable logistic regression for the receipt of tissue-based biomarker tests.

	HRR-clustered multivariable logistic regression		
	OR	95% CI	*P*
**Residency**			
Metro	Ref.	Ref.	
Urban	0.78	0.72 to 0.85	<.0001
Rural	0.82	0.73 to 0.91	.0003
Unknown	1.00	0.88 to 1.14	.9
**Age group**			
66-70 years	Ref.	Ref.	
71-75 years	0.88	0.87 to 0.90	<.0001
76-80 years	0.69	0.67 to 0.70	<.0001
≥81 years	0.32	0.31 to 0.33	<.0001
**Race/Ethnicity**			
White	Ref.	Ref.	
Black	0.82	0.77 to 0.88	<.0001
Hispanic	0.80	0.73 to 0.88	<.0001
Asian	0.90	0.79 to 1.02	.1
Other	0.86	0.75 to 0.98	.02
Unknown	1.10	1.05 to 1.14	<.0001
**US Region**			
South	Ref.	Ref.	
West	1.01	0.86 to 1.18	.9
Midwest	0.93	0.79 to 1.09	.4
Northeast	0.88	0.75 to 1.03	.1
**Dual eligibility for Medicaid**			
Yes	Ref.	Ref.	
No	1.49	1.41 to 1.56	<.0001
**Disability as the original reason for Medicare entitlement**			
Yes	Ref.	Ref.	
No	1.43	1.38 to 1.49	<.0001
**Year of prostate cancer diagnosis**			
2019	Ref.	Ref.	
2020	1.37	1.32 to 1.42	<.0001
2021	1.87	1.78 to 1.95	<.0001
2022	2.43	2.31 to 2.56	<.0001
2023	2.69	2.53 to 2.87	<.0001
**Number of chronic conditions**			
0	Ref.	Ref.	
1	1.18	1.13 to 1.23	<.0001
2	1.27	1.23 to 1.32	<.0001
≥3	1.35	1.30 to 1.40	<.0001

Abbreviations: OR = odds ratio; 95% CI = 95% Confidence Interval.

## Discussion

This is the first national study to assess geographic variability in the use of tissue-based biomarkers for prostate cancer within a full, national Medicare claims data source. We found a large degree of variability in use across US hospital referral regions with approximately twenty times higher utilization in the top hospital referral regions. Rural patients and urban patients (defined as those in mid-sized communities) were both less likely receiving tissue-based biomarker tests compared to those who lived in major population centers. Although testing rates increased steadily each diagnosis year since 2019, patients in less urbanized counties were consistently less likely to receive tissue-based biomarker testing. Independently, Black and Hispanic patients were also less likely to undergo testing.

We identified statistically significant differences in tissue-based biomarker test utilization across metro, rural, and urban counties. These findings are in line with other research showing geographic cancer care disparities across multiple cancer types. Overall, rural patients tend to show higher cancer incidence^24^ and mortality^25^ rates. Patients in rural regions also demonstrate differences in screening, diagnosis, and treatment practices.^25^^,^^26^ Geographic disparities in prostate cancer care remain understudied, despite evidence of poorer routine diagnostic workup in rural areas such as fewer diagnostic biopsy cores or multidisciplinary consultations.^3^

Prostate cancer diagnostics, however, have grown increasingly comprehensive in recent years.^6^ These include advanced imaging, genetic, and pathologic techniques.^27^ While these tools hold promise for improving diagnostic accuracy and enabling better treatment decisions, they could also exacerbate disparities if disseminated unequally.^28^ It is important to note that the tests investigated in our study are not routinely recommended, and their contribution to clinical decision-making remains debated. We therefore specifically refer to “advanced diagnostics,” which clinicians should understand have limitations. Nevertheless, tissue-based tests are frequently used, as our study highlights, and patients in rural and urban counties were up to 22% less likely to receive testing compared to those in metro counties.

A benefit of tissue-based biomarker tests is their ability to be performed as an add-on to routine biopsies or prostatectomy specimens. After initial histological assessment, formalin-fixed paraffin-embedded tissue can be re-analyzed.^29^ This method eliminates the need for additional patient visits and reduces the burden of long travel times. Unlike resource-intensive imaging requiring on-site facilities and appointments, tissue-based biomarker testing needs less infrastructure and can additionally be performed at reference laboratories.^30^ Such remote testing opportunities could particularly benefit patients from underserved and rural areas.

However, the increasing frequency and complexity of advanced pathologic diagnostics may exacerbate rural-urban disparities in accessing high-quality cancer care. Notably, our data showed increasing odds of tissue-based biomarker tests in patients diagnosed from 2020 to 2023. Similarly, Bologna et al. observed significantly increased tissue-based biomarker test utilization between 2011 and 2021 in de-identified claims data.^31^ Although testing rates and odds increased over diagnosis time among patients with prostate cancer, geographic disparities between metro and less urbanized counties persisted in our analysis. Advanced and more frequent diagnostic testing, from traditional pathology to molecular and genetic analyses, requires both specialized infrastructure and expertise for high-quality care delivery.^32^ Nass et al. proposed solutions to address present and future challenges. Their recommendations included digital solutions, artificial intelligence, telemedicine involvement, multidisciplinary collaborations, and adoption of clinical decision support tools. These approaches could also help bridge the rural-urban divide.^13^

Our study also revealed a large variability in test utilization at a regional level, independent of rural/urban classifications. With testing rates ranging from 2.4% to a maximum of 42.7% across hospital referral regions, these findings underscore regional practice-level differences. Our results align with previous work that found variation in tissue-based testing early adoption across hospital referral regions during the initial post-coverage period.^33^ This regional analysis suggests that differences in resource availability and clinical care conditions may influence the uptake of novel prognostic technologies, underscoring the need to investigate patient-level determinants of testing.^33^ While our analysis addressed the patient-level patterns, the observed regional variations could also potentially be attributed to physician-level factors, particularly urologists' perceptions regarding its potential utility for risk stratification. Regional differences in testing rates might be influenced by local practice patterns and provider preferences rather than healthcare access disparities. Recent evidence shows that practice-level factors such as financial incentives, physician organization, and the structure of the local healthcare market contribute to treatment variation in prostate cancer, especially when clinical uncertainty is high.^16^ Interestingly, the observed utilization pattern contrasts with current guideline recommendations: While for example germline genetic testing is strongly recommended for a broad patient population,^34^ it is performed in only 1%-2% of patients with prostate cancer.^35^^,^^36^ Conversely, tissue-based biomarker testing shows higher utilization rates with up to 42.7% use in specific hospital referral regions, despite not being routinely recommended in any clinical prostate cancer scenario in current guidelines,^37^^,^^38^ which supports practice-level differences in approaching tests. Nevertheless, these tests could help inform treatment decisions for selected patients with localized disease.^34^^,^^39^ Tissue-based biomarker tests analyzed in our study are prognostic for outcomes including adverse pathology after radical prostatectomy,^40^ biochemical recurrence,^41^ metastases,^6^ and prostate cancer mortality.^7^

Prior research has shown that tissue-based biomarker tests influence treatment decisions. In a prospective study of 158 predominantly white patients, Badani et al. reported that test utilization increased active surveillance recommendations by 24%.^42^ Conversely, a randomized trial by Carbunaru et al. showed that testing led to a significant decrease in active surveillance recommendations from urologists.^43^ These findings are particularly interesting as the study primarily included Black (70%) and Hispanic (12.5%) patients and were confirmed in another randomized clinical trial that also predominantly included Black patients.^43^^,^^47^ Black and Hispanic patients face known disparities in prostate cancer care, including differences in disease stage at diagnosis, screening rates, and diagnostic workup.^44^^,^^45^ Our study found that Black and Hispanic patients were less likely to undergo tissue-based biomarker tests. Importantly, these tests perform equally well across racial groups^40^ and could serve as an important tool in tailoring treatment for different populations. Recent evidence suggests shared decision making can mitigate racial disparities in prostate cancer screening. Without shared decision-making discussions, Black and Hispanic males were less likely to undergo screening compared to White males.^48^ Tissue-based biomarker tests could support a shared decision-making process by providing additional prognostic information.^7^ For example, some of the tests examined in our study might improve the identification of suitable candidates for active surveillance.^6^ Although long-term studies have demonstrated the safety of active surveillance across ethnic groups, its utilization remains lower among minoritized populations.^50^^,^^51^ Implementing these tests could help reduce the observed racial disparities in active surveillance adoption and potentially address the higher rates of disease progression and local therapy observed among Black patients under active surveillance.^49^

In our study, certain covariates closely associated with rurality influenced the likelihood of tissue-based biomarker tests. Medicare disability enrollment and dual Medicare/Medicaid eligibility, both indicators of lower socioeconomic status,^52^^,^^53^ were associated with lower tissue-based biomarker test utilization. Social determinants of health are known to influence prostate cancer diagnostic workup and likely contribute to the rural-urban divide.^26^^,^^54^ A study by Leapman et al. demonstrated that sociodemographic factors, including dual Medicare/Medicaid eligibility, largely explained racial disparities in prostate MRI.^55^ Furthermore, rural patients tend to be older, have poorer general health, and less access to prevention services, which all contribute to poorer health outcomes.^56^

### Strengths and limitations

A key strength of the study lies in its comprehensive analysis of tissue-based biomarker testing patterns through national Medicare claims data for patients with prostate cancer. While the database lacks clinical parameters such as PSA values and tumor characteristics, this key limitation is mitigated by the substantial regional variation observed. The 20-fold difference in testing rates across hospital referral regions is unlikely to be explained by clinical parameters alone, indicating a potential influence of non-clinical factors on test use. It is also important to note that because our data come exclusively from Medicare claims, our study population consists only of insured individuals. This limitation may lead to an underestimation of testing disparities—especially since rural populations tend to have higher rates of uninsurance.^26^ Further, some miscoding or overlap with other molecular tests may have affected test identification. Still, CPT codes for Next generation sequencing and related diagnostics have been widely used to assess testing prevalence in other cancers.^46^^,^^33^ Finally, because our analysis is restricted to Medicare beneficiaries and utilizes Medicare’s standard racial categories, the findings may not be generalizable to younger patients (<65 years) or to populations described by more nuanced ethnic classifications.

## Conclusion

Our analysis of Medicare claims data reveals stark geographic variability in use of tissue-based biomarker tests in prostate cancer care. Although clusters in hospital referral regions were observed, patients in less urbanized areas face constant reduced access to this diagnostic tool, highlighting a rural-urban divide and practice-level differences in prostate cancer care. Further, racial disparities also emerged in testing utilization. By increasing access to tissue-based biomarker tests in underserved areas, healthcare providers could potentially provide more personalized treatment strategies and ultimately improve outcomes for rural patients with prostate cancer.

## Supplementary Material

pkaf051_Supplementary_Data

## Data Availability

For this study, we accessed Medicare claims data through a licensed agreement with the Centers for Medicare & Medicaid Services (CMS). In accordance with the standard CMS data use agreement, we are not permitted to share the data directly.
