# Supplementary material for: Disparities in tissue-based biomarker testing among US Medicare beneficiaries with prostate cancer
Source: JNCI Cancer Spectr. 2025 May 16;9(4):pkaf051. doi: 10.1093/jncics/pkaf051 (PMC12212051; doi:10.1093/jncics/pkaf051)
Supplement: pkaf051_Supplementary_Data [file pkaf051_supplementary_data.docx]

**Supplementary Appendix**

Supplementary Figure 1, Flow chart of the study population selection process of Medicare beneficiaries newly diagnosed with prostate cancer 2019-2023.

Final cohort of beneficiaries

(n = 749,202)

Exclusion of beneficiaries without resident zip code and/or female beneficiaries (n = 6,964)

Exclusion of beneficiaries with Medicare Part A and Part B enrollment < 1 year and/or age < 66 years old (n = 450,007)

Exclusion of beneficiaries with a preexisting Prostate Cancer Diagnosis in 2018 (n = 1,169,181)

Beneficiaries with ICD-10 Prostate Cancer diagnosis C61 between 2018 and 2023 (n = 2,375,354)

Supplementary Table 1, Code set for tissue-based biomarker tests,

|  | CPT/HCPCS Codes |
| --- | --- |
| CPT/HCPCS Codes tissue-based biomarker tests, at least one code or a combination of these codes was required to identify patients. | 81445, 81455, 81479, 81541, 81542, 0047U, 0057U, 0021U |

Abbreviations: Current Procedural Terminology (CPT)/Healthcare Common Procedure Coding System (HCPCS) codes

Supplementary Table 2, Rural-Urban Continuum Codes by the US Department of Agriculture and the used definition of metro, urban and rural areas

| 2023 Rural Urban Continuum Codes | | | Study classification |
| --- | --- | --- | --- |
| Original Classification | **Code** | **Description** | **Category** |
| Metro counties | 1 | ≥1 million population | Metro |
|  | 2 | 250,000-1 million population |  |
|  | 3 | <250,000 population |  |
| Nonmetro counties | 4 | ≥20,000, metro-adjacent | Urban |
|  | 5 | ≥20,000, not metro-adjacent |  |
|  | 6 | 5,000-20,000, metro-adjacent |  |
|  | 7 | 5,000-20,000, not metro-adjacent | Rural |
|  | 8 | <5,000, metro-adjacent |  |
|  | 9 | <5,000, not metro-adjacent |  |


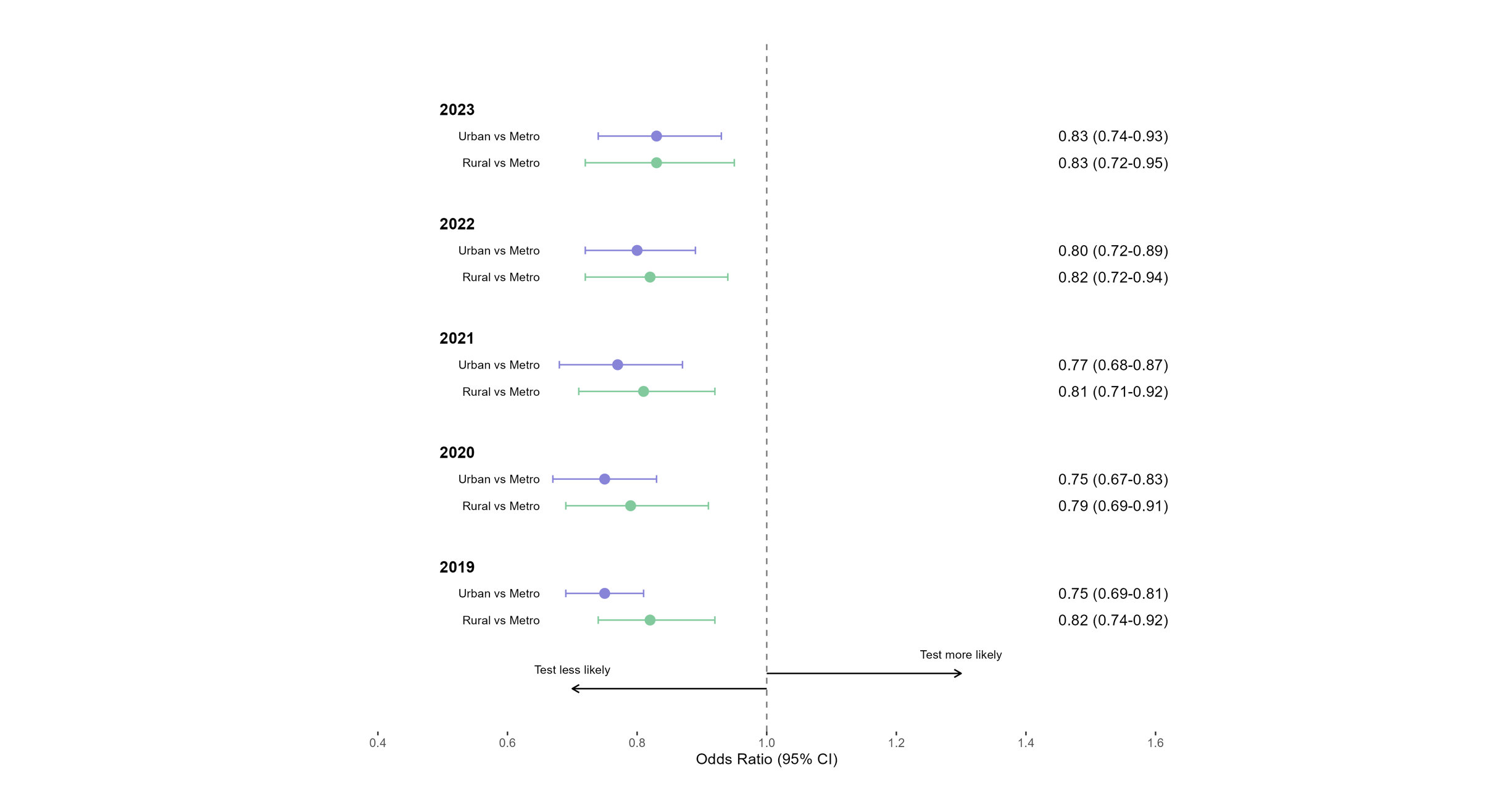


Supplementary Figure 2, Forest plot of the Interaction term analysis between residency and year of diagnosis in the multivariable regression model clustered at HRR level for patients newly diagnosed with prostate cancer 2019-2023. Throughout the years patients were diagnosed with prostate cancer, patients were less likely to undergo tissue-based biomarker tests in rural and urban counties compared to metro counties.

STROBE Statement

“Disparities in Tissue-based Biomarker Testing Among U.S. Medicare Beneficiaries with Prostate Cancer”

|  | Item No | Recommendation | Page No |
| --- | --- | --- | --- |
| **Title and abstract** | 1 | (*a*) Indicate the study’s design with a commonly used term in the title or the abstract | 3 |
|  |  | (*b*) Provide in the abstract an informative and balanced summary of what was done and what was found | 3,4 |
| Introduction | | | |
| Background/rationale | 2 | Explain the scientific background and rationale for the investigation being reported | 5,6 |
| Objectives | 3 | State specific objectives, including any prespecified hypotheses | 5,6, |
| Methods | | | |
| Study design | 4 | Present key elements of study design early in the paper | 7 |
| Setting | 5 | Describe the setting, locations, and relevant dates, including periods of recruitment, exposure, follow-up, and data collection | 7,8 |
| Participants | 6 | (*a*) Give the eligibility criteria, and the sources and methods of selection of participants. Describe methods of follow-up | 7,8 |
|  |  | (*b*) For matched studies, give matching criteria and number of exposed and unexposed |  |
| Variables | 7 | Clearly define all outcomes, exposures, predictors, potential confounders, and effect modifiers. Give diagnostic criteria, if applicable | 8,9 |
| Data sources/ measurement | 8 | For each variable of interest, give sources of data and details of methods of assessment (measurement). Describe comparability of assessment methods if there is more than one group | 7, 8, 9, |
| Bias | 9 | Describe any efforts to address potential sources of bias | 7 |
| Study size | 10 | Explain how the study size was arrived at | 7 |
| Quantitative variables | 11 | Explain how quantitative variables were handled in the analyses. If applicable, describe which groupings were chosen and why | 9 |
| Statistical methods | 12 | (*a*) Describe all statistical methods, including those used to control for confounding | 9 |
|  |  | (*b*) Describe any methods used to examine subgroups and interactions |  |
|  |  | (*c*) Explain how missing data were addressed |  |
|  |  | (*d*) If applicable, explain how loss to follow-up was addressed |  |
|  |  | (*e*) Describe any sensitivity analyses |  |
| Results | | |  |
| Participants | 13 | (a) Report numbers of individuals at each stage of study—eg numbers potentially eligible, examined for eligibility, confirmed eligible, included in the study, completing follow-up, and analysed | Suppl. Figure 1 |
|  |  | (b) Give reasons for non-participation at each stage |  |
|  |  | (c) Consider use of a flow diagram |  |
| Descriptive data | 14 | (a) Give characteristics of study participants (eg demographic, clinical, social) and information on exposures and potential confounders | 10 |
|  |  | (b) Indicate number of participants with missing data for each variable of interest |  |
|  |  | (c) Summarise follow-up time (eg, average and total amount) |  |
| Outcome data | 15 | Report numbers of outcome events or summary measures over time | 10 |

| Main results | 16 | (*a*) Give unadjusted estimates and, if applicable, confounder-adjusted estimates and their precision (eg, 95% confidence interval). Make clear which confounders were adjusted for and why they were included | 10,11 |
| --- | --- | --- | --- |
|  |  | (*b*) Report category boundaries when continuous variables were categorized |  |
|  |  | (*c*) If relevant, consider translating estimates of relative risk into absolute risk for a meaningful time period |  |
| Other analyses | 17 | Report other analyses done—eg analyses of subgroups and interactions, and sensitivity analyses | 11 |
| Discussion | | | |
| Key results | 18 | Summarise key results with reference to study objectives | 12 |
| Limitations | 19 | Discuss limitations of the study, taking into account sources of potential bias or imprecision. Discuss both direction and magnitude of any potential bias | 16 |
| Interpretation | 20 | Give a cautious overall interpretation of results considering objectives, limitations, multiplicity of analyses, results from similar studies, and other relevant evidence | 12, 13,14, 15, 16, 17 |
| Generalisability | 21 | Discuss the generalisability (external validity) of the study results | 15, 16 |
| Other information | | | |
| Funding | 22 | Give the source of funding and the role of the funders for the present study and, if applicable, for the original study on which the present article is based | 2 |
